# New Composite Material for Masonry Repair: Mortar Formulations and Experimental Studies

**DOI:** 10.3390/ma14040912

**Published:** 2021-02-15

**Authors:** Walid Deboucha, Ibrahim Alachek, Jean-Patrick Plassiard, Olivier Plé

**Affiliations:** 1 Université Savoie Mont Blanc, CNRS, LOCIE, 60 rue du lac Léman, 73000 Chambéry, France; walid.deboucha@esitc-caen.fr (W.D.); ibrahim.alachek@univ-smb.fr (I.A.); olivier.ple@univ-smb.fr (O.P.); 2 Laboratoire deRecherche ESITC Caen, COMUE Normandie Université, 14610 Epron, France

**Keywords:** mortar-based composite, masonry, textile-reinforced mortar (TRM), tensile tests, bond tests, low carbon solution

## Abstract

The need for retrofitting existing masonry structures is progressively becoming more important due to their continuous deterioration or need to meet the current design requirements of Eurocodes. Textile-Reinforced Mortar (TRM) composite systems have emerged as a sustainable repair methodology suitable for structure retrofitting. Nevertheless, their mechanical performance is still far from being fully investigated. This paper presents an experimental study on the tensile and bond behaviors of a new mortar-based composite consisting of mineral additives, blended cement mortar, and stainless-steel grid. Three different mineral additives (silica fume, fly ash, and blast furnace slag), in binary and ternary systems were used. The experimental study included uniaxial tensile coupon testing on composite specimens and bond tests on composite material applied to clay-brick substrate. The results obtained with the different textile-reinforced cement-based mortars were compared and are discussed here. It was found that, for mortar formulations containing mineral additives—such as fly ash or blast-furnace slag—with high tensile and bond strengths, an adequate adherence between the constituents was obtained. The developed mortar presents mechanical performances equivalent to traditional mortars without additives. The study contributes to the existing knowledge regarding the structural behavior of TRM and promotes the development of a low impact carbon cementitious matrix.

## 1. Introduction

In the rehabilitation of buildings, the repair and strengthening of structures are of vital importance for their safeguarding and durability. Currently, there is an increasing trend to repair structures in order to preserve, rather than demolish them. In the last decade, the application of a composite material to the surface of structural elements has been successfully used worldwide for strengthening and seismic retrofitting of concrete and masonry structures. Selected case studies of real applications of externally bonded reinforcement in the construction field can be found in [1]. Epoxy resin-based composites, such as fibre reinforced polymers (FRPs), are one of the most commonly used techniques to repair or reinforce structural elements [2,3,4]. This technique, however, is not recommended for applications in masonry structures for several reasons, such as the low resistance to fire [5] and poor transpiration of the substrate due to the presence of epoxy matrix [6]. Recently, a new technique for the repair and strengthening of masonry structures, namely textile reinforced mortar (TRM), was proposed. TRM consists of fibre grids embedded in a mortar-based matrix. Carbon and glass fibres, aramid textiles, steel cords, basalt, and even natural fabrics are used in TRM [5,6]. Compared with FRPs, TRM improves resistance to fire, provides better adherence for materials, has higher tensile strength, ensures an adequate chemical compatibility with the substrate, adequate efficiency from a health perspective, and has a better capacity for energy dissipation during significant or accidental events like earthquakes [6,7,8,9,10]. In addition to these advantages, TRM is more suitable for masonry structures thanks to easier application on rough surfaces, lower costs, and a shorter installation time [5,6,11,12].

Owing to these major advantages, TRM is gaining considerable attention in research, with several experimental studies on TRM being published during recent years [6,11,12,13,14,15,16,17,18]. However, further research is needed to provide a better understanding of the effects of various parameters, such as the fabric type and cement matrix, on the mechanical behaviour of TRM.

In the majority of studies in the literature, a commercial repair mortar was generally used in the TRM [16,19,20,21]. However, the commercial repair mortar is not often appropriate for repairing structures with TRM composite materials where some difficulties may be encountered during its application, such as the high rate of losing workability or the low viscosity [20]. Indeed, to date, no recommendation or European standard relating to the composition of a mortar has been adopted for this type of repair. Hence, investigations on the composition of cement-based matrix are needed. Only a few recent studies have focused on the effect of mortar matrices on mechanical behaviour, i.e., tensile and shear bond behaviour of TRM [6]. De Santis and de Felice [6] investigated the tensile behaviour of composites-based mortars comprising two different fabrics (glass–aramid and steel) and five different mortar matrices that were prepared with (a) pure cement, (b) natural kaolin and bauxite fired at 1000–1200 °C, (c) pozzolan and polypropylene microfibres, and (d) polymer modified cement mortar. It was found that the tensile behaviour of the composites-based mortar studied depended on the mechanical properties of both the matrix composition and the fabric, as well as the fabric-to-matrix bond. They concluded that the stiffer and stronger matrices (matrices containing cement in their formulations) lead to higher stiffness in both the un-cracked and cracked stages and also a better interlocking with the textile. Butler et al. [13] investigated the durability of TRM made with multi-filament yarns of AR-glass and different mortar matrices. Their results indicated that the performance enhancement of TRM relied on the alkalinity and hydration process of the mortar. Moreover, low alkalinity mortar, i.e., mortar prepared with mineral additives (fly ash and microsilica), was useful in controlling the performance of TRM as a result of the good bonding between the mortar and fabric (AR glass). Indeed, the use of mineral additives as cement replacement in mortar influences the hydration reactions and the formation of solid phases in the fabric-matrix interface. According to Butler et al. [13], the addition of pozzolanic additives to Portland cement results in the formation of a stratum of thin Calcium silicate hydrate (CSH) phases instead of portlandite (Ca(OH)_2_) leading to a good bonding capacity and hence, greater stiffness. More recently, Dong et al. [22] examined the tensile behaviour of glass textile embedded in cement mortar prepared with different levels of cement replacement by flash ash (42% and 65%). It was found that the addition of fly ash to cement at higher replacement level (65%) produced a better deformation capacity than the one obtained in the case of cement replaced by fly ash at lower replacement level (42%). Signorini et al. [23] researched the tensile behaviour of TRM made with glass textile coated by silica-based mineral and lime mortars. Tow lime mortars with large and small macro-pores were used to prepare the composite. It was found that the use of mortar with large macro-pores as embedding matrix decreased the bonding capacity between fabric and mortar matrix.

In addition to improving the bonding between mortar and fabric, the use of mineral additives as cement replacement in the mortar should help reduce CO_2_ emissions during cement production. In fact, it was observed that the addition of up to 70% of mineral additives to Portland cement provided a stable, durable, economic, and cleaner environment, by reducing CO_2_ emissions and recycling industrial waste materials [24,25]. In this context, this article presents experimental investigations on the effect of a matrix with mineral additives on the uniaxial tensile and bond behaviours of a new mortar-based composite for use as externally bonded reinforcement systems. Silica fume, fly ash and blast furnace slag were used in the preparation of mortar matrices. A stainless-steel grid was used in this investigation as a textile fabric. First, using a combination of silica fume, fly ash and blast furnace slag in binary and ternary systems, different mortar matrix compositions were prepared, by varying the replacement level, the volume of the binder and the water-to-binder ratio so as to improve the workability of fresh mortar and enhance the mortar strength. Five mortar matrices were then selected to prepare the composite, with the first being the reference case without an additive. The mechanical properties of the mortar (flexural behaviour, compression strength) were evaluated. The tensile behaviour of the stainless-steel grid-reinforced matrix (SSGRM) composite was also analysed to determine the performance of the newly-developed composite material. In addition, small-scale shear bond tests were conducted to characterize the SSGRM-to-brick bond behaviour of the five mortar formulations tested. These results allowed us to define the most appropriate formulation out of the five that were tested. Finally, the effects of anchorage length on bond strength and the failure mode of the bond test specimens were investigated and are discussed here for the chosen formulation.

The main purpose of this study is to provide a better understanding of fundamental behavioural mechanics of mortar-based composite and to provide quantitative data on the effect of the use of mineral additives in the mortar formulation on the strength and stiffness of TRM composite, and on the bond strength of composite to masonry substrate. This paper particularly reports specific economical mortar formulations for the repair of masonry structures which could be easily mixed and equivalent in performance to the better proprietary materials. As a result of the investigations, a mortar formulation was developed which allows for adequate exploitation of the material properties. This formulation is based on a combination of an artificial Portland cement CEM I 52.5, fine sand, fly ash, and plaster retarder.

## 2. Materials and Test Methods

### 2.1. Stainless-Steel Grid

The textile fabric used in this study is a stainless-steel grid, made of 6 mm spaced cords. The diameter of the steel cords is 1 mm and the area of cords per unit width is 112.87 mm^2^/m. Tensile tests were carried out on the grid to identify its characteristics [20]. In their study [20], three identical coupons with dimensions of 250 mm clear length and 100 mm width were tested. The test was carried out using a universal testing machine of 120-kN capacity. The specimens were gripped to the testing machine using two aluminium plates (with dimensions of 125 mm long and 100 mm width) that were glued to their ends using a structural adhesive. An extensometer was mounted at the centre of the coupons over a gage length of 160 mm to measure the tensile strain. The load was applied monotonically under displacement control at a rate of 1 mm/min up to failure. All grid coupons failed due to rupture of the fibres at the central region of the coupon within the gauge length of the extensometer. The grid exhibited a strong ductility, with the strength maintained even for strains of up to 36%. The average mechanical properties of the stainless-steel grid are listed in Table 1.

### 2.2. Cement Mortars

As mentioned earlier, five mortar matrices prepared with the combination of silica fume, fly ash, and blast furnace slag in binary and ternary systems were selected. Blast furnace slag and fly ash were used as cement replacement to improve the workability, strength, and durability [24,26], as well as the bonding between mortar and fabric. Moreover, silica fume was used to compensate for the lower strength in the early ages induced by blast furnace slag and fly ash. The replacement level of Portland cement by mineral additives was based on previous studies [24,27], where it was found that there was no loss in the mechanical performance of mortar or concrete when the Portland cement was replaced by 20% (by weight) mineral additives. The mix proportions of the mortar matrices considered in this study are displayed in Table 2.

The applied mixes were self-compacting with a water-to-cement ratio w/c of approximately 0.3 and a maximum aggregate size, d_max_, of 2 mm. An artificial Portland cement CEM I 52.5 produced by LAFARGE with a commercial name Durabat X-Trem was used to prepare the mortar. The chemical admixture applied was a polymer-based superplasticizer which reduces the surface tension of water during mixing, thus allowing the concrete consistency to increase without additional water. Using such high range water-reducing admixtures gives way to the reduction of the water-cement ratio, which in turn increases the strength of the mortar.

The properties of fresh mortar, e.g., slump and workability, were investigated in order to make appropriate adjustments to obtain a suitable mortar mix for certain cases. The results of these tests, however, are not presented here as they are beyond the scope of this study. Only the selected mixtures and their mechanical properties are presented in this paper.

For the selected matrices, various specimens were prepared, cast, and cured under water at room temperature. Compression and bending flexural tests were performed at 7 and 28 days of curing time. For each curing time, three mortar test pieces (16 × 4 × 4 cm^3^) were assigned for the 3-points bending flexural test and six 40 mm-edge cubes for the compression test. The tests were conducted following the European Standards (EN 196-1:2016) [28]. The specimens were demoulded after 24 h from casting, and then were immersed and stored in a water tank at 20 ± 1 °C. The specimens were then removed from the tank at various intervals for testing. The compression tests were performed using an automatic compression testing machine with a loading rates of 2500 N/s. The same machine was used to perform the bending tests. For these tests, the mortar prism was placed in the testing machine with one side face on the supporting rollers and with its longitudinal axis normal to the supports. The clear distance between supporting rollers was about 100 mm. The load was applied on the opposite side face of the prism through a loading roller and was increased smoothly at the rate of 50 N/s until fracture.

### 2.3. Tensile Test: Specimen Geometry and Preparation

**Figure 1:**
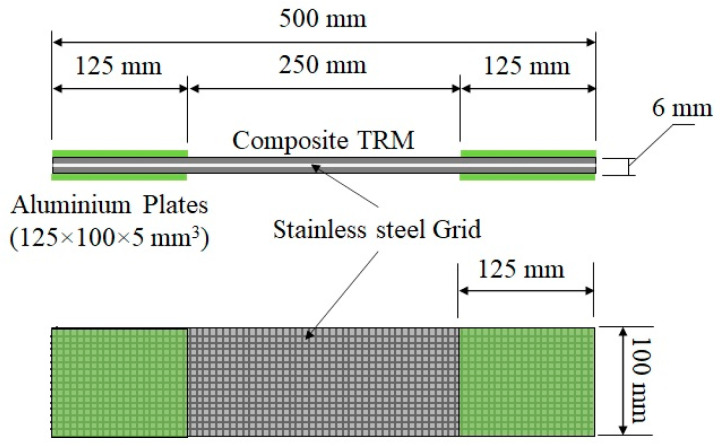
Tensile specimen geometry.

The axial behaviour of tensile composite specimens made of a stainless-steel grid embedded in a mortar-based matrix (SSGRM) was studied experimentally. The tests were similar to those proposed in the RILEM TC 232-TDT [29]. The specimens have an overall cross section of 100 mm × 6 mm (±0.5) and a length of 500 mm, as shown in Figure 1. The tests were performed after 7 days of curing by means of a testing machine equipped with a 120 kN electric actuator, under monotonic increasing load with displacement control at a constant stroke rate of 0.017 mm/s. During the tests, the load and machine displacement were recorded in a data acquisition system (sampling rate of 10 Hz). Only the side of the specimen that was cast against the bottom of the mould was analysed due to its smooth appearance. Three identical specimens were tested for each mortar formulation.

**Figure 2:**
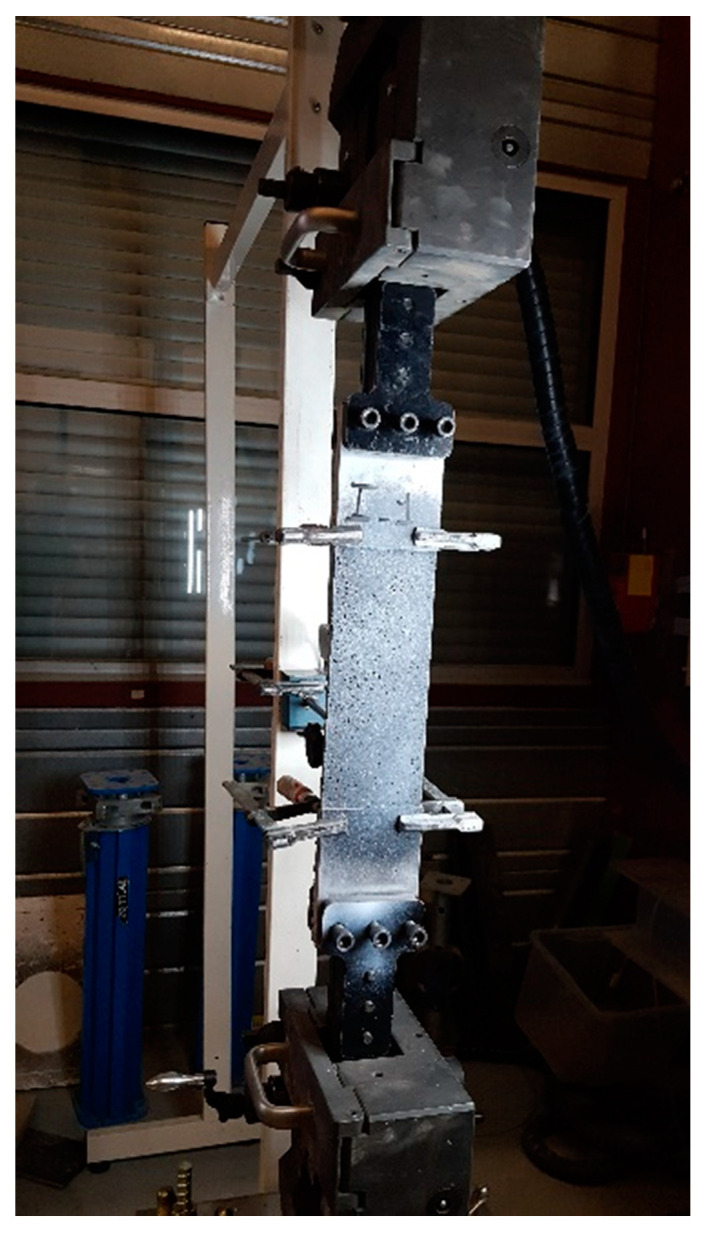
Tensile test setup.

In order to guarantee a uniform load transfer and to prevent stress localization within the clamping regions, four aluminium plates (125 mm × 100 mm × 5 mm) were glued using an epoxy structural adhesive (Sikadur 31-EF) at the loaded ends of specimens. During the tests, the tabs were clamped within the wedges of the testing machine. The pressure was adjusted to prevent slippage between the clam and the specimen. Two clamps were also used to avoid delamination between the specimens and the lower ends of the aluminium plates [15], as shown in Figure 2.

### 2.4. Bond Test: Specimen Geometry and Preparation

In recent years, externally bonding cement-based composite has been widely used to upgrade and retrofit masonry structures [6,9,11,12,15,17]. One of the critical failure modes of shear strengthened structures is the debonding failure of the externally bonded composites. This unfortunate failure had led to extensive research on the bond behaviour between composite materials and masonry in the past two decades aiming to expand the knowledge and understanding of the shear bond behaviour and the factors influencing the adherence and bonding mechanism. Bond behaviour is typically investigated experimentally using the “shear bond” test of TRM-to-concrete bonded joints, where a TRM is applied to a masonry substrate and subjected to a tension force. This test is important for evaluating the composite action between the assembled materials which is a determining factor for performance. The test procedure and the specimen geometry are described in detail in the following paragraphs.

The composite specimen tested herein is a six-phase material consisting of textile reinforcement (stainless steel-grid), mortar matrix, hollow clay brick, an interface between textile reinforcement and cementitious matrix and the interface between the cementitious matrix and brick. The force transferred from the mortar matrix to the reinforcement is governed by the quality of the bond between the reinforcement and the matrix, while the force transferred from the brick to the mortar matrix is governed by the brick surface roughness, as well as the interaction and compatibility between the cementitious matrix and the clay brick [9,15]. The quality of these interfaces represents a critical factor to be considered in order to achieve an effective retrofitting system. The aim of this test was to characterize the shear bond behaviour of the TRM to the substrate.

**Figure 3:**
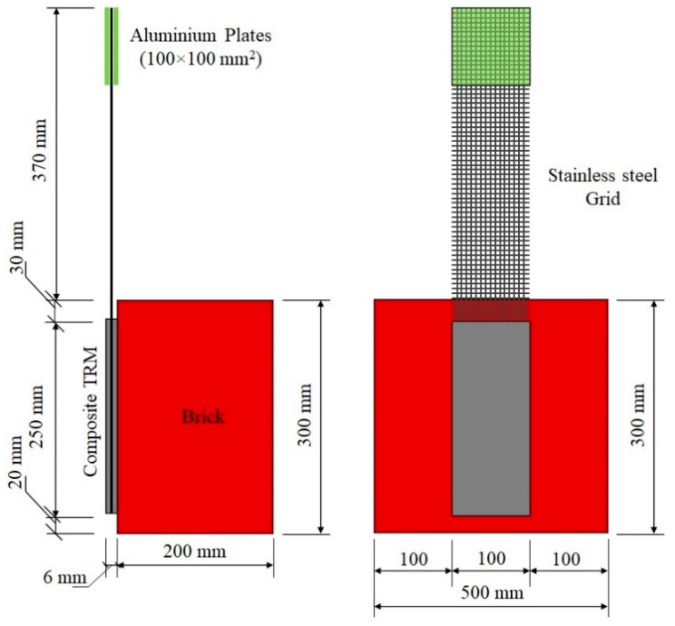
Bond specimen geometry.

The shear bond behaviour of the SSGRM applied on a clay brick was experimentally evaluated. The tests were similar to those proposed in the RILEM TC 250-CSM [30]. The geometry of the bond specimens is shown in Figure 3. The following procedure was adopted for the preparation of specimens. The contact surface of clay brick was first humidified. Next, a 3 ± 1 mm-thin mortar layer was applied on the brick surface and the steel grid was pressed slightly into the fresh mortar for approximately 30 s. During this process, the mortar protruded through the openings of the grid slightly upward. After that, the second mortar layer was applied to achieve a total thickness of approximately 6 mm. The specimens were cured under ambient laboratory conditions (ca. 15–20 °C and 50–60 % relative humidity) and the tests were performed after 28 days of curing by means of a testing machine equipped with a 120 kN electric actuator, under monotonic increasing load with displacement control at a constant stroke rate of 0.017 mm/s. During the tests, the load and machine displacements were recorded in a data-acquisition system (sampling rate of 10 Hz) and two linear variable differential transducers (LVDTs with 30 mm stroke), mounted at the loaded end, were used to measure the relative displacements between the free part of the SSG and the clay brick. The displacement is expressed as the mean value of the two LVDTs in the following. Three repetitions were performed for each mortar formulation.

As in the tensile test, the same gripping method was used in the bond test to hold the unbonded grid. For all the tests, a laser level was used along the in-plane and the out-of-plane directions, to adjust the position of specimens in order to avoid misalignments in the load application.

### 2.5. Additional Tests: Determination of Anchoring Length

**Figure 4:**
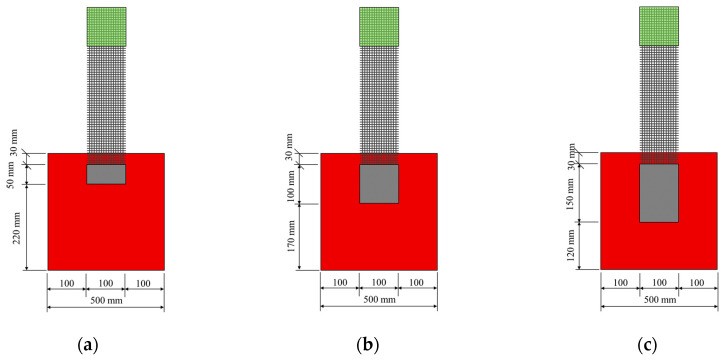
Bond specimen geometries for the anchoring length of: (**a**) 50 mm, (**b**) 100 mm, and (**c**) 150 mm.

The bond tests, presented in Section 2.4, have the advantage of comparing the effect of several formulations. Thus, the most appropriate formulation can be selected. Nevertheless, the bond test does not indicate the anchoring length that is required in order to prevent a failure by debonding [15]. This parameter is called optimal anchoring length in the following. It is of fundamental importance, especially if a local repair [20] or a repair by strips is considered. To characterize the bond behaviour of the SSGRM-Brick of the five selected mortar formulations, an anchoring length of 250 mm was used, as recommended in the RILEM TC 250-CSM [30]. This is considered as the reference test in the following. A new series of bond tests was carried out, in which the anchorage length was varied. As described previously, three identical samples were created for each anchorage length tested. The smallest anchorage length considered was 50 mm. This length was then increased for each trio of samples by increments of 50 mm, until the exploitation ratio obtained converged with that of the reference bond test. Samples with anchorage lengths of 50, 100, and 150 mm were built; all three geometries are presented in Figure 4.

## 3. Results and Discussion

### 3.1. Mortar Specimens

**Figure 5:**
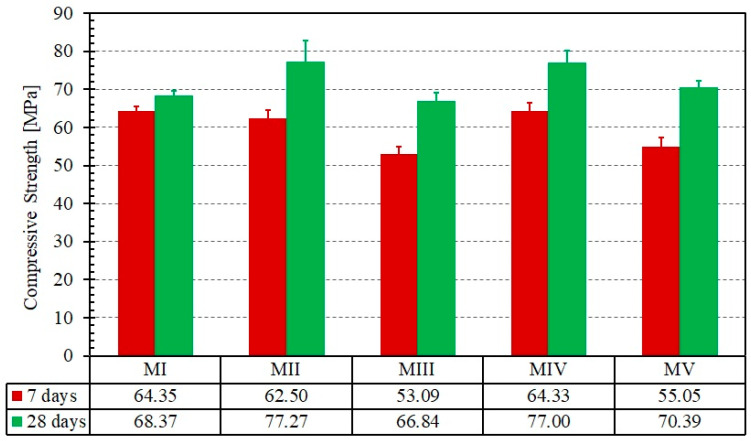
Compression test results for the five mortar formulations at different curing times. Error bars represent the standard deviation of the mean.

**Figure 6:**
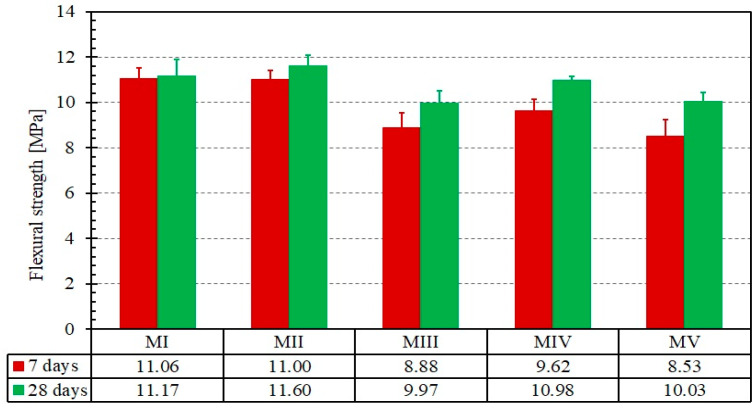
Flexural test results for the five mortar formulations at different curing times. Error bars represent the standard deviation of the mean.

The mechanical properties of the mortar (cubic compressive strength, flexural tensile strength) after 7 and 28 days of curing time were quantified and presented in Figure 5 and Figure 6, respectively.

First, each of the five mortars exhibited a low discrepancy in both tensile and compression strengths, which allowed for a sound comparison of the results. This confirms the results obtained in [24,27] where a similar strength was recorded for mortars prepared using Portland cement with or without mineral additives, i.e., blast furnace slag, at a replacement level of 20%. Regarding the development of compressive strength, the mortar matrix prepared with blast-furnace slag (MII) and the mortar matrix prepared with both blast-furnace slag and silica fume (MIV) were the most suitable for the preparation of composite and for the repair of masonry walls from a mechanical and workability perspective. For these two formulations, a suitable consistency and slowly rate of losing workability were noticed. The mortar matrix prepared with blast-furnace slag (MII) exhibited the highest tensile strength of the four mortars that included additives. Its strength is about the same as that of MI, for which no additive was used. It is worth noting that both the compressive and tensile strengths are twice the values of a commercial repair mortar used in previous works [20]. The high tensile strength should help in achieving a higher cracking strength as will be discussed in Section 3.2.

At this point, the second and fourth formulation is the most suitable one for the preparation of composite and repairing of masonry walls from a mechanical and workability point of view. However, bond and tensile tests of the composite are required for an objective comparison of the five formulations. The selection will be based on the bonding capacity between the steel-grid and the mortar matrix as well as the adherence between the composite (fabric and mortar) and the clay brick.

### 3.2. Tensile Test Results and Discussions

The results are discussed based on the load versus jack-displacement response. Ultimate load value in the tensile test is defined here as the load obtained at the end of the test (at 60 mm jack displacement). It is worth noting that the reinforcement did not reach its maximal tensile strength in any of the cases and testing could have continued; however, these tests were terminated after the total delamination of the mortar matrix, i.e., pull-out of reinforcement without further crack development. Another justification for not extending the test lies in the fact that the large strains obtained would probably not be encountered in a masonry wall subjected to a damage loading.

**Figure 7:**
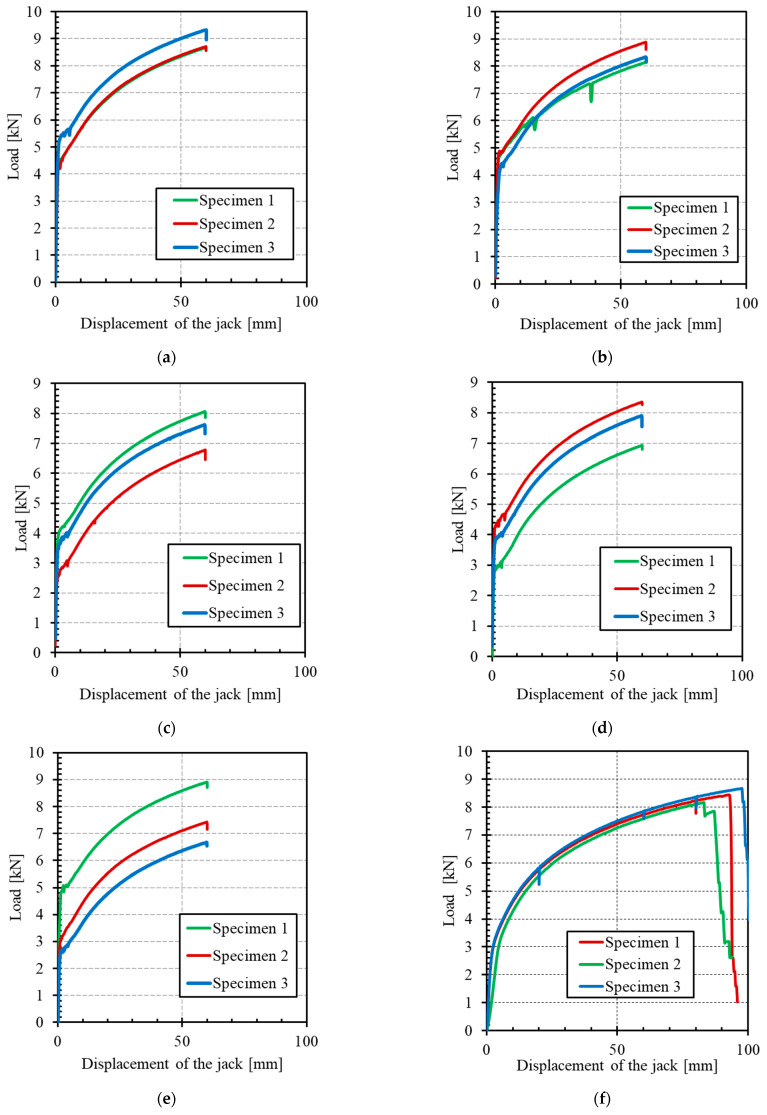
Load–displacement curves for the tensile specimens: (**a**) formulation MI, (**b**) formulation MII, (**c**) formulation MIII, (**d**) formulation MIV, (**e**) formulation MV, and (**f**) stainless-steel grid (SSG).

**Figure 8:**
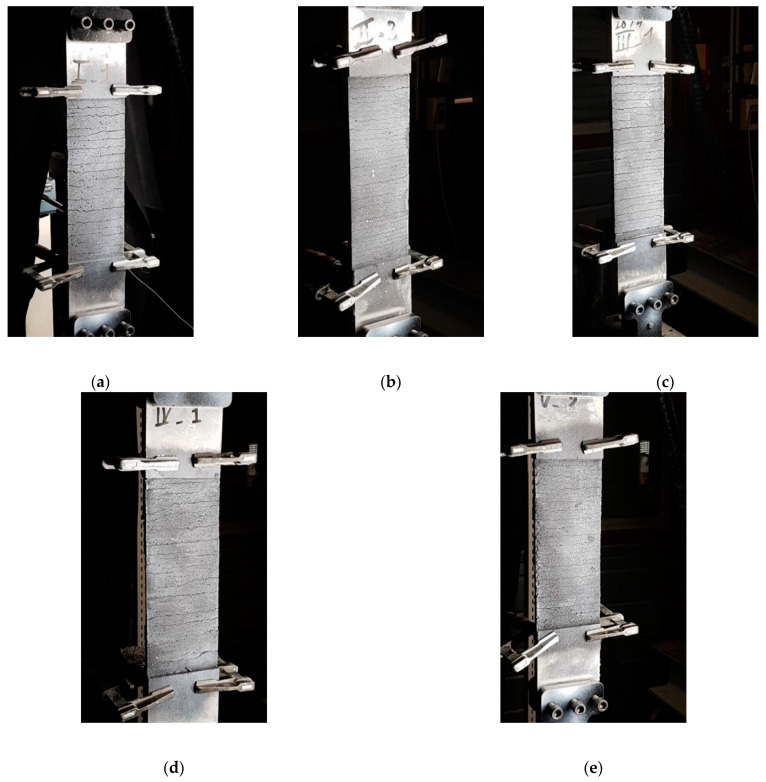
Cracking pattern of the tensile specimens for the different mortar formulations: (**a**) formulation MI, (**b**) formulation MII, (**c**) formulation MIII, (**d**) formulation MIV, (**e**) formulation MV.

A summary of the tensile test results is presented in Table 3. The load–jack-displacement relationships obtained for the five different mortar formulations and that of the stainless-steel grid alone are shown in Figure 7. Figure 8 illustrates the cracking patterns of the specimens tested.

The curves obtained reflect the typical trilinear behaviour of TRM materials, consisting of a first linear-elastic branch, a second multiple crack formation phase and a third region dominated by the textile response [15,31]. However, the distinctiveness of the current composite is the absence of brittle failure that is encountered with FRP, as already noted elsewhere [32]. The ultimate tensile strength of the composite specimens, despite the differences in mortar formulation, was found to be close to that of the stainless steel-grid alone (in the order of 8 kN). The scatter of results and the low values of the stress level at the end of the tests for some specimens, especially in the third branch, can be explained by the misalignment of the textile layer (parasite flexure, out of plan movement, etc.), the alignment of end plates, the matrix heterogeneity, and the local stress concentration.

As reported by De Santis and De Felice [6], the mechanical properties of mortar mainly affect the initial non-cracking behaviour and cracking strength (load level just before the appearance of the first crack) and have negligible influence on the tensile strength and crack stiffness of the composite. In other words, the first crack strength F_cr_ is mainly a function of the matrix strength and quality and thus its value is an indication of the matrix durability performance, while the ultimate strength F_u_ is mainly a function of the interface bond between the matrix and fabric and of the fabric strength, therefore its value gives an indication of the composite performance. Regarding the cracking strength, the experiments showed that the mortar formulations MI and MII exhibit the highest values of F_cr_. This could be explained through the high tensile strength exhibited by MI and MII, as mentioned in Section 3.1. The first cracking strength F_cr_ is related to the serviceability limit state (SLS) conditions and should be interpreted with care, since one may interpret the formation of cracks as an exceedance of the SLS requirements, while others may interpret the crack formation and propagation until a certain width as an acceptable SLS condition. This load level can then be used as an indicator of the composite performance, with a high value of F_cr_ signifying a high-performance composite and delayed development of cracks. The ratio of the first crack strength F_cr_ to the bending tensile strength in Figure 6 was calculated for each formulation. This ratio is relatively stable and ranges between 0.33 and 0.41. This result indicates that the first crack of the composite is principally linked to the mortar properties and not to the interaction between the mortar and the grid.

Concerning the crack pattern, the development of cracks observed during tensile tests on composites was characterized by the appearance of transversal cracks perpendicular to the direction of the applied load. Cracking first occurs when the tensile strength of the matrix is reached (theoretically), after which multiple cracking of the matrix occurs in the strain-hardening stage (stage II). The number of cracks and their spacing and width were found to be similar for the five mortar-formulations tested in this study. Compared to other mortar, i.e., lime or fly ash mortars, De Santis and de Felice [6] and Dong et al. [22] found that the use of cement mortar instead of lime mortar or cement mortar with a high replacement level of fly ash (65%) produced a better stiffness in cracking. Considering each formulation independently, the progression of the strength was very similar after the first crack from one test to another, since the gap between the three curves remained constant for the direction of stage II (Figure 7). Moreover, the force-vs-displacement curves were much smoother than those obtained in a previous study with the same stainless-steel grid [20]. One main reason is the lesser strain rate applied during the current study, while several jolts were noted in previous experiments. Nevertheless, the curves in Figure 7 appear to be much more regular, compared to other TRM made from textiles such as basalt [33], aramid [34], glass-aramid [15], steel cords, and carbon [35], among others. This last observation could be very useful in terms of the safety coefficients to be used for design purposes.

### 3.3. Bond Tests

The results of the bond tests for the different mortar formulations are summarized in Table 4. Both the failure mode and the ultimate strength of each mortar formulation are presented. In the following sub-sections, the tests results will be discussed in detail.

#### 3.3.1. Failure Modes

**Figure 9:**
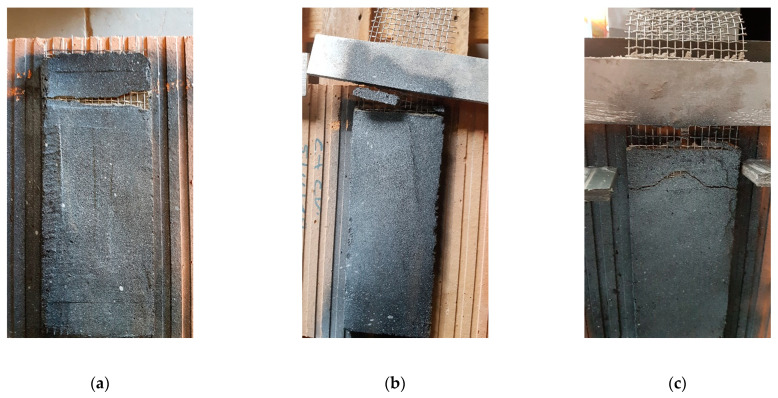
Failure mode of the specimens of the mortar formulations: (**a**) MI, (**b**) MII, and (**c**) MIII.

**Figure 10:**
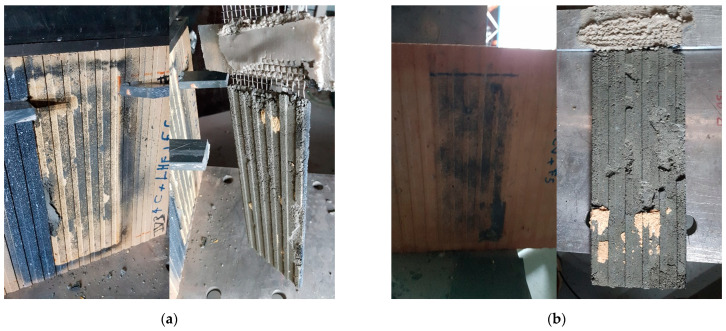
Failure mode of the specimens of mortar formulations: (**a**) MIV and (**b**) MV.

The failure modes observed in the tested specimens can be classified into two types:

- Mixed failure mode: tensile rupture of the steel grid out of the bonded area with mortar splitting at the loaded end. The failure was initiated by the formation of a single transversal crack on the top part of the bonded area (at an early stage of loading). This crack opened further at the end of the test and the top 1–3 cm of the matrix detached completely from the clay brick. The development of this crack can be explained by the textile elongation within the mortar matrix during the elastic behaviour stage. The rest of the interface was visibly intact, and no cracks/separation was observed. Finally, the failure was ended by rupture of the longitudinal steel cords progressively. This might be attributed to the bonding capacity between fabric and mortar matrix as well as the adherence between composite (fabric and mortar) and clay brick [6,13]. Examples of this failure mode are shown in Figure 9.
- Debonding of TRM from the clay substrate without peeling off parts of the clay brick (as shown in Figure 10). Here, the TRM composite remains almost intact after failure and detaches from the substrate as a single body.

The first failure mode occurred in all specimens of the mortar formulations MI, MII and MIII, whereas the second occurred in all specimens of the formulation MIV and in two specimens of the formulation MV.

#### 3.3.2. Numerical Results

**Figure 11:**
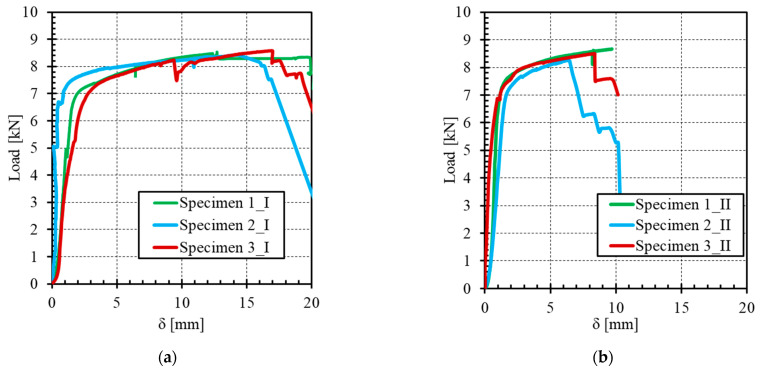

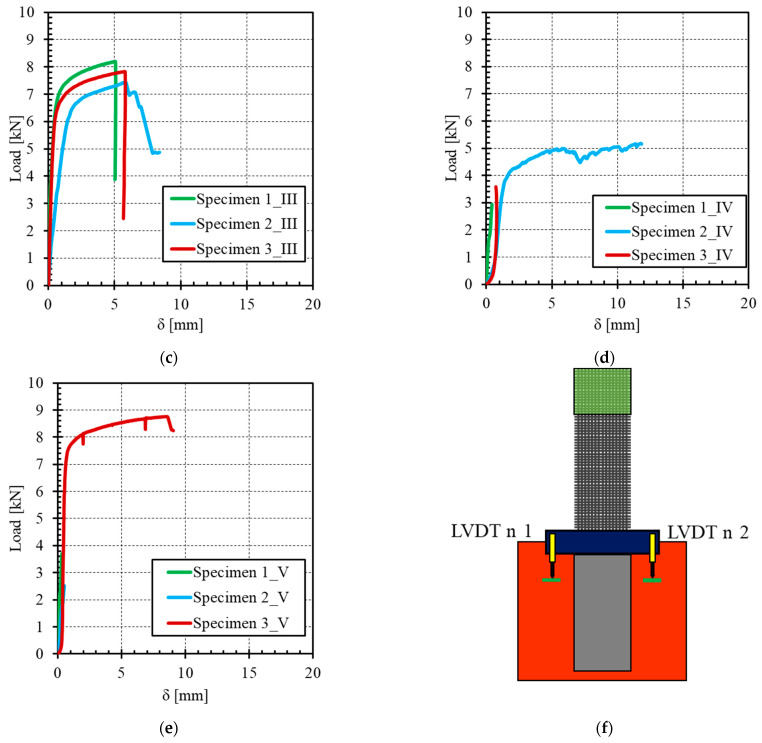
Load–relative displacement between SSG and clay brick for the different mortar formulations: (**a**) formulation MI, (**b**) formulation MII, (**c**) formulation MIII, (**d**) formulation IV, (**e**) Formulation MV, and (**f**) locations of the LVDTs.

The axial load–slip curves of the different mortar formulations are plotted in Figure 11, while Table 4 summarizes the results of the bond tests.

The stress transfer mechanisms (load-displacement curves) occurring between the SSG stainless steel grid (SSG) and clay brick are typically divided into two stages, as shown in Figure 11.

- Stage I–Elastic: the linear response signifies the perfect bond between the SSGRM composite and brick.
- Stage II–Non-linear: this stage depends on the failure mode.
  1. Failure mode 1: nonlinear behaviour of the SSG (ductility) followed by brutal rupture of the steel cords.
  2. Failure mode 2: partial debonding starts and spreads along the bonded length with successive debonding until entire embedded length is debonded. This behaviour stage was not observed because of the high stroke rate. The SSGRM was brutally separated from the clay brick.

From the experimental results, the higher peak load was obtained with the mortar-formulations MI and MIII but for different values of maximum slip (14.64 mm for formulation MI and 8.16 mm for formulation MIII). The larger relative displacements recorded between the SSG and the brick are related to the high ductility and deformability of the SSG, and they can also be related to a possible slippage of the SSG within the mortar matrix. For the formulation MIII, the peak load was slightly lower than that of the first two formulations, but the slip value decreased significantly, which reflects a better quality of adherence between the SSG and mortar on the one hand and between the SSGRM and the brick on the other hand. For the last two mortar formulations MIV and MV, the values of peak load and slip were very low compared with those obtained with formulations MI-MIII. In the case of MIII, the amelioration of the adherence between SSG and mortar could be explained through the pozzolanic reaction where the fly ash can react with portlandite in presence of water to form more CSH and thus reduces the macro-pores. This results in high bonding capacity as reported by Signorini et al. [23] and Butler et al. [13].

Concerning the exploitation ratio of textile strength (equal to the maximum axial stress in the textile attained in the bond test ($f_{b}$) divided by the tensile strength in the textile derived from a tensile test ($f_{t}$)), its value was found to be higher for the three first mortar-formulations (MI, MII and MIII) (up to more than 100%), whereas for formulations MIV and MV, the exploitation ratio was less than 50%.

On the basis of these last results combined with those shown in Table 2, it can be assumed that the silica fume degrades the efficiency of the bond between the TRM and the clay brick. Silica fume was used to compensate the low strength induced by blast furnace slag and fly ash at early mortar ages. However, a faster increase in strength at early stages can induce a loss of adhesion [36]. To compensate for this phenomenon, the use of a geopolymer binder is recommended [37] and could represent an improvement to the current study.

The bond tests highlighted that formulations MIV and MV were not appropriate, as early debonding was noticed. The failure load was almost one third of that of formulations MII and MIII. Among these two remaining formulations, the choice was made to focus on formulation MII. This choice is justified by the better results obtained during the aforementioned tensile tests, in terms of tensile strength as well as cracking strength. Thereafter, experiments were based on formulation MII exclusively.

### 3.4. Determination of Anchoring Length

The failure mode of specimens prepared using formulation MII was mixed, as indicated in Table 4, but most of the bond surface was still in place at the end of the tests. This means that the anchoring length of 250 mm is sufficient to allow a full exploitation of the tensile strength of composite material. This was possible thanks to the adequate bond strength and anchoring length between the matrix and the clay brick. However, to identify the optimal anchoring length for which the load carrying capacity is maximal and no further increase can be found beyond this value, bond tests on specimens with different anchoring lengths are needed.

**Figure 12:**
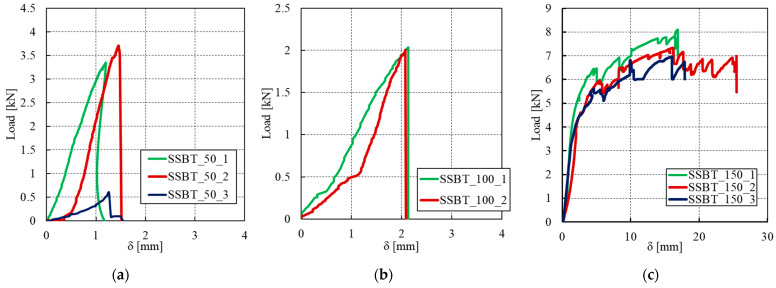
Load–relative displacement between SSG and clay brick of the different anchoring lengths: (**a**) 50 mm, (**b**) 100 mm, and (**c**) 150 mm.

The average results of all the tested specimens are presented in Figure 12. Here, “SSBT” denotes the shear bond test, “50, 100, and 150” denote the anchoring length in millimetres, while the last number denotes the sample number. The mean peak load, mean maximum relative displacement (slip) measured by the LVDT, exploitation ratio, and ultimate shear of each anchoring length are summarized in Table 5. The ultimate shear stress was calculated by dividing the ultimate load at failure by the bonded area.

**Figure 13:**
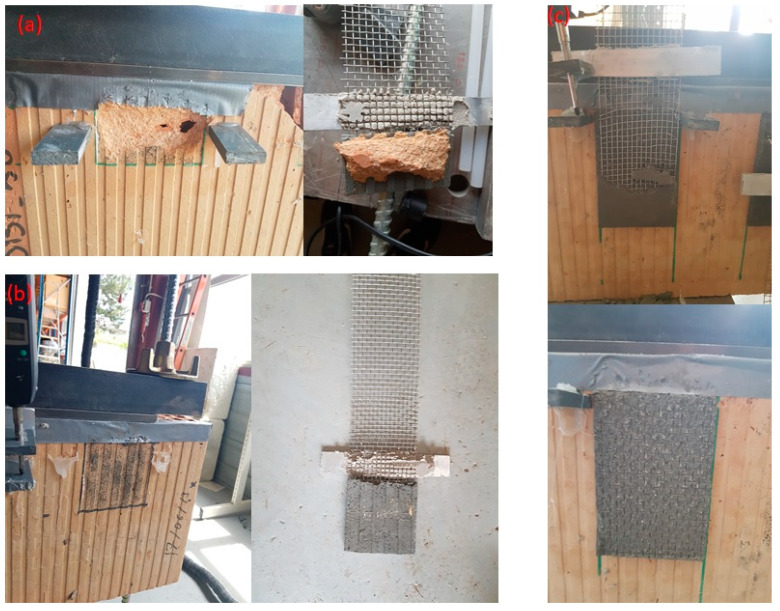
Failure modes of specimens with an anchorage length of (**a**) 50 mm, (**b**) 100 mm, and (**c**) 150 mm.

All the specimens exhibited an initial uncracked behaviour, associated with a stiff response, as shown in Figure 12. The behaviour is almost brittle for the two smallest anchoring lengths of 50 mm and 100 mm. One sample of each anchorage length did not even reach a significant strength. For the 50 mm anchorage length, it broke directly by debonding at the substrate-matrix interface in only a few seconds after applying the load. The same observation was made at the very start of one test for the 100 mm anchorage length, so that only two samples could be considered for these anchorage lengths. For other two specimens with an anchorage length of 50 mm, a cohesive failure within the clay brick was observed. A chunk of the brick attached to the SSGRM was generally found after failure, as shown in Figure 13a. This mode of failure proves that the matrix has allowed for a full transmission of efforts between the two substrates, in other words, the matrix has provided a stronger adhesion bond than the shear strength of the brick. Regarding specimens with 100 mm anchorage length, Figure 13b shows that failure occurred at the brick/matrix interface with splitting of the clay-brick. This may be explained by the low adhesion between the matrix and the brick due to the geometrical imperfections or by several factors occurring during the preparation of the specimens, such as the presence of small air bubbles and surface preparation (humidification). This is confirmed by the low exploitation ratio obtained with this anchorage length (Table 5). By contrast, the samples with the 150 mm anchoring length exhibited a non-linear behaviour with high ductility, corresponding to the generation of multiple cracks in the composite. The results from the different specimens with this anchorage length are almost repeatable and the exploitation ratio is close to 1.0, which is also quite close to the value obtained with the highest anchorage length of 250 mm. For all the specimens, the failure mode observed from the bond tests was cohesive by debonding at the textile-matrix interface, as shown in Figure 13c. The rupture of the specimens was by shearing in the matrix close to the textile-matrix interface. Indeed, a transversal crack in the matrix near the loaded end of composite was observed first. This crack led to detachment of the matrix from the textile and some matrix chunks fell from the composite. The matrix detachment continued expanding and ultimately became wide enough to lead to the separation of the textile and the matrix. A thick mortar layer was kept with the brick after specimen failure, indicating a high adhesion between the matrix and the brick. This observation is in agreement with the findings of previous experimental studies on SSGRM [15].

The results of the bond tests performed on SSGRM showed that the anchorage length may have a significant effect on the failure mode and the exploitation ratio of the composite. As a result, it was found that the anchoring length of 150 mm is sufficient to ensure the efficiency of the current TRM.

## 4. Conclusions

In this article, experimental investigations on the mechanical behaviour of an externally bonded (EB) new mortar-based composite made of a stainless-steel grid (SSG) embedded in a cement-based matrix are presented, with the aim of contributing to the development of an economic cement mortar for the repair of masonry structures, with low environmental impact, and for establishing an adequate and practically applicable retrofitting technique.

It is important to note that the results obtained in this study might not apply generally to all types of stainless-steel grid-reinforced matrix (SSGRM) and that the conclusions should be considered within the context of the materials tested in this study, namely the textile reinforced mortar components as well as the clay brick. On the basis of the results obtained and the observations made during the experimental tests, the following conclusions may be drawn:

- As a result of this experimental study, mortar matrices, based on the combination of silica fume, fly ash, and blast furnace slag in binary and ternary systems, were developed which are equivalent in performance to the better mortars of this same general type.
- Blast furnace slag and fly ash can be used as cement replacement to improve the workability, strength, and durability, as well as the bonding between mortar and fabric.
- Tensile tests showed that the SSGs allow for a good stress distribution in the cement-based matrix, with a diffuse cracking mode.
- SRGRM composite displayed a non-linear behaviour with high ductility before failure, which ensures the flexibility-deformability (energy dissipation) of the composite.
- Results of the bond tests indicated that formulations involving silica fume had satisfactory inherent strength but a very low quality of bond on the clay brick.
- High tensile and bond strengths can be achieved and, a good adherence between the constituents of the assembly (brick, mortar and SSG) can also be obtained, with the mortar formulations containing fly ash or blast-furnace slag.
- Increasing the anchorage length resulted in higher load-carrying capacity of bond specimen. An optimal anchorage length of approximately 150 mm for which a high exploitation ratio of the SSG was found.

The experimental results presented herein represent a contribution to the investigation of the mechanical behaviour and performance of EB mortar-based systems for the strengthening/retrofitting of masonry. Further studies and in depth analyses of the effects of anchorage length are needed to better evaluate the effects of each admixture on the adhesion between mortar and brick and on the performance of the mortar, which will help draw general practical conclusions for the design of this type of structure.

## Figures and Tables

**Table 1:** Stainless Steel Grid (SSG) tensile properties.

Type	Acronym	$\mathrm{Tensile}\;\mathrm{Strength}\;f_{t}\;\left(MPa\right)$	Elastic Modulus E (GPa)	$\mathrm{Ultimate}\;\mathrm{Strain}\;\varepsilon_{u}\;\left(\%\right)$
Steel	SSG	670.0 ± 19.5	27.6 ± 12.1	36.4 ± 2.9

**Table 2:** Matrix design per m^3^, water-to-binder ratio 0.3.

Component	Quantity [kg/m^3^]						
	Mineral Admixtures					Chemical Admixtures	
Matrix	Cement (CEM I 52.5)	Fly ash	Silica fume	Blast-furnace slag	Fine aggregate d_max_ < 2 mm	Superplasticizer	Water
MI	800	-	-	-	1325.1	25	223
MII	640	-	-	160	1319.1	25	223
MIII	640	160	-	-	1315.7	25	223
MIV	640	-	40	120	1308.8	25	223
MV	640	120	40	-	1306.2	25	223

**Table 3:** Mean results of the tensile tests (average value of the three individual values ± standard deviation).

Formulation	F_cr_ (kN)	F_u_ (kN)	σ_c_ (N/mm^2^)	σ_f_ (N/mm^2^)
MI	4.68 ± 0.51	8.90 ± 0.38	14.83 ± 0.63	708.29 ± 30.11
MII	4.65 ± 0.2	8.28 ± 0.53	13.80 ± 0.88	658.93 ± 41.95
MIII	3.31 ± 0.69	7.30 ± 0.65	12.17 ± 1.09	581.00 ± 51.86
MIV	3.65 ± 0.7	7.45 ± 0.46	12.41 ± 0.76	592.75 ± 36.26
MV	3.50 ± 1.18	7.45 ± 1.45	12.41 ± 2.41	592.51 ± 115.12

σ_c_: composite stress corresponds to the stress level in the composite which equal to the applied load divided by the cross section of the specimen. σ_f_: fibre stress corresponds to the stress level in the textile which is equal to the applied load divided by the area of the fibres (product of the number of longitudinal steel cords in the direction of loading and the section of steel cord).

**Table 4:** Mean results of the bond tests (average value of the three individual values ± standard deviation).

Formulations	Peak Load (kN)	Slip (δ) (mm)	$\mathrm{Exploitation}\;\mathrm{Ratio}\;\eta =f_{b}/f_{t}$	Mean Ultimate Shear Stress (MPa)	Failure Mode
MI	8.52 ± 0.08	14.67 ± 2.13	0.96 ± 0.01	0.34 ± 0.00	Mixed
MII	8.53 ± 0.23	8.16 ± 1.67	1.03 ± 0.03	0.34 ± 0.01	Mixed
MIII	7.83 ± 0.37	5.42 ± 0.38	1.07 ± 0.05	0.31 ± 0.01	Mixed
MIV	3.26 ± 0.46	0.58 ± 0.21	0.44 ± 0.06	0.13 ± 0.02	Debonding
MV	3.11 ± 0.83	0.41 ± 0.12	0.42 ± 0.11	0.12 ± 0.03	Debonding

**Table 5:** Summary of experimental and analytical results of the bond tests (average value of the three individual values ± standard deviation).

Anchorage Length (mm)	Peak Load (kN)	Slip (δ) (mm)	$\mathrm{Exploitation}\;\mathrm{Ratio}\;\eta =f_{b}/f_{t}$	Mean Ultimate Shear Stress (Mpa)	Failure Mode
50	2.55 ± 1.70	1.30 ± 0.14	0.31 ± 0.21	0.51 ± 0.34	CB/DB
100	2.02 ± 0.02	2.11 ± 0.04	0.24 ± 0.00	0.20 ± 0.00	DB
150	7.47 ± 0.58	16.34 ± 0.45	0.90 ± 0.07	0.50 ± 0.04	TF/DI
250	8.53 ± 0.23	8.16 ± 1.67	1.03 ± 0.03	0.34 ± 0.01	TF

CB: Cohesive within the brick (peeling of the brick surface); DB: debonding at substrate-matrix interface; TF: tensile failure of textile out of the composite; DI: debonding at textile-matrix interface.

## Data Availability

The data presented in this study are available on request from the corresponding author. The data are not publicly available due to the confidentiality order.
